# Structural and Functional Insights into the Catalytic Inactivity of the Major Fraction of Buffalo Milk Xanthine Oxidoreductase

**DOI:** 10.1371/journal.pone.0087618

**Published:** 2014-01-31

**Authors:** Kaustubh S. Gadave, Santanu Panda, Surender Singh, Shalini Kalra, Dhruba Malakar, Ashok K. Mohanty, Jai K. Kaushik

**Affiliations:** 1 Animal Biotechnology Centre, National Dairy Research Institute, Karnal, Haryana, India; 2 BTIS Subcentre, National Dairy Research Institute, Karnal, Haryana, India; La Trobe University, Australia

## Abstract

**Background:**

Xanthine oxidoreductase (XOR) existing in two interconvertible forms, xanthine dehydrogenase (XDH) and xanthine oxidase (XO), catabolises xanthine to uric acid that is further broken down to antioxidative agent allantoin. XOR also produces free radicals serving as second messenger and microbicidal agent. Large variation in the XO activity has been observed among various species. Both hypo and hyper activity of XOR leads to pathophysiological conditions. Given the important nutritional role of buffalo milk in human health especially in south Asia, it is crucial to understand the functional properties of buffalo XOR and the underlying structural basis of variations in comparison to other species.

**Methods and Findings:**

Buffalo XO activity of 0.75 U/mg was almost half of cattle XO activity. Enzymatic efficiency (*k*_cat_/*K*_m_) of 0.11 sec^−1^ µM^−1^ of buffalo XO was 8–10 times smaller than that of cattle XO. Buffalo XOR also showed lower antibacterial activity than cattle XOR. A CD value (Δε_430 nm_) of 46,000 M^−1^ cm^−1^ suggested occupancy of 77.4% at Fe/S I centre. Buffalo XOR contained 0.31 molybdenum atom/subunit of which 48% existed in active sulfo form. The active form of XO in buffalo was only 16% in comparison to ∼30% in cattle. Sequencing revealed 97.4% similarity between buffalo and cattle XOR. FAD domain was least conserved, while metal binding domains (Fe/S and Molybdenum) were highly conserved. Homology modelling of buffalo XOR showed several variations occurring in clusters, especially close to FAD binding pocket which could affect NAD^+^ entry in the FAD centre. The difference in XO activity seems to be originating from cofactor deficiency, especially molybdenum.

**Conclusion:**

A major fraction of buffalo milk XOR exists in a catalytically inactive form due to high content of demolybdo and desulfo forms. Lower Fe/S content and structural factors might be contributing to lower enzymatic efficiency of buffalo XOR in a minor way.

## Introduction

Xanthine oxidoreductase (XOR) catalyzes the oxidative hydroxylation of hypoxanthine to xanthine and subsequently xanthine to uric acid [1]. XOR occurs as a homodimer; each subunit with independent catalytic activity and molecular mass of 147 kDa contains one molybdenum, one FAD and two 2Fe-2S (Fe/S) centres [1], [2]. In mammals, XOR exists in two interconvertible forms, xanthine dehydrogenase (XDH; EC 1.1.1.204), which predominates *in vivo*, and xanthine oxidase (XO; EC 1.1.3.22). Both forms of the enzyme can reduce molecular oxygen to produce superoxide anion (•O_2_^−^), although only XDH can reduce the preferred electron acceptor NAD^+^ to produce superoxide anion and H_2_O_2_ instead. Apart from oxidation of hypoxanthine and xanthine, XOR can also catalyze the hydroxylation of a wide range of N-heterocyclic and aldehyde substrates [1]. The uric acid and its oxidative product allantoin in ruminants and lower vertebrates act as potent antioxidants and free radical scavengers, and thereby providing protection against oxidative damage [3], [4]. On the other hand, XOR is also involved in the synthesis of reactive oxygen species (ROS) and reactive nitrogen species (RNS) for killing microbes [5], [6]. XOR also regulates expression of other genes like cyclooxygenase-2 [7]. Because of these activities, XOR has been implicated in various pathophysiological conditions like Type I diabetes [8], vascular oxidative stress [9], postischemic tissue injury, respiratory and cardiovascular disorders [10] characterized by increased oxidative stress conditions. XOR expression is highly regulated by tumorigenesis [11], hopoxia [12], mechanical stress, endotoxins and cytokines [6], [10], [13].

In the context of milk synthesis, XOR plays critical role in the development, pregnancy, and lactation in mammals. In the case of milk fat globule membrane, XOR has been recognized as a major protein component [14] and implicated in the process of milk lipid secretion [15], [16]. Knockout of XOR gene in mice led to defective enveloping of milk fat droplets with the apical epithelial plasma membrane resulting in premature involution of the mammary gland [17].

XOR is an evolutionary conserved gene; nevertheless, biochemical properties of milk XOR differed significantly among the species. Human XOR showed an oxidase activity of 0.07 U/mg, while oxidase activity of XOR from cattle, sheep and goat has been measured to 1.4–1.8 U/mg [18], [19], 0.69 U/mg [18] and 0.27 U/mg [20], respectively. Incidentally higher XO activity results in the elevated production of uric acid causing arthritis like condition in human [21]. On the other hand, decreased XOR activity could result in xanthinuria [22]. Higher level of consumption of meat and seafood has also been associated with an increased risk of gout, while dairy food is associated with a decreased risk of gout [23]. XOR present in animal milk has been detected in the blood stream of human consumers [24], [25]. Due to debilitating nature of XOR mediated pathophysiological conditions research efforts have been directed for the development of inhibitors against XOR [21], [26]–[28]. On the other hand, XOR also showed growth promoting effect and almost 50% decrease in scours in animals [29]. XOR is a major milk fat globule membrane (MFGM) protein and, incidentally, buffalo milk contains higher fat percentage in comparison to cattle's milk. In spite of the fact that XOR plays a significant role in the regulation of the cellular redox potential, innate immune system and various pathophysiological conditions in humans and animals, little is known about the XOR from buffalo milk [30], [31]. In south Asia, sizable amount of milk comes from buffalo, and constitutes ∼55% of the total milk production in India [32]. Given the multifarious effects of XOR on human health, it was crucial to understand the biochemical and physicochemical properties of buffalo milk XOR. We cloned and characterized XOR from buffalo milk and showed that though it has highest sequence similarity with cattle sequence, biochemical properties of XOR from these species are markedly different. The oxidase activity of buffalo milk XOR was almost half of the cattle XOR, while the enzymatic efficiency was almost 10 times lower than cattle milk XOR. Cofactor composition of XOR also differed significantly between the two species. The large difference in the XO activity could be originating from varying cofactor deficiency and minor structural differences in XOR of various species.

## Materials and Methods

All the chemicals and buffers used in the purification and estimation of various cofactors were of analytical or equivalent grade. Xanthine, sodium bicine, sodium iodide, sodium sulfite, sodium sulphide, thiourea, dimercaptotoluene, mercaptoacetic acid, acrylamide and glycine were purchased from Sigma Chem. Co., USA, while rest of the chemicals wherever not mentioned were procured from Merck India.

### Purification of XOR

The purification protocol was similar to that described by Godber *et al.* [19], [33]. Fresh unpasteurized buffalo (*Bubalus bubalis*, Murrah breed) and cattle (*Bos indicus*, Sahiwal breed) milk was procured from dairy farm of National Dairy Research Institute at Karnal, India and protein purification was initiated within 30 min of milking. Cream was separated from 2.0 litre fresh milk by centrifugation at 2000×g for 30 min at 4°C. The separated cream was redissolved in 1.0 litre buffer (0.2 M K_2_HPO_4_, 5.0 mM DTT with 1.0 mM EDTA) by vigorous stirring. The cream mixture suspension was centrifuged at 3000×g for 30 min at 4°C and the supernatant was filtered through layered muslin cloth. The filtrate was slowly mixed with 15% (v/v) butanol prechilled at −20°C followed by addition of ammonium sulfate (15 g/100 ml) with continuous stirring for an hour allowing the precipitation of protein, which was removed by centrifugation at 8000×g for 20 min at 4°C. Further 20 gm/100 ml of ammonium sulfate was slowly added to the supernatant with continuous stirring that resulted in the appearance of brownish precipitate that was removed by centrifugation at 9500×g for 30 min at 4°C. The precipitate was redissolved in 25 mM 4-morpholineethanesulfonic acid (MES) containing 1.0 mM EDTA at pH 6.0 and the suspension was dialyzed against 3.5 litre of the same solution. Any remaining precipitate was removed by centrifugation at 10,000×g for an hour at 4°C. The filtered sample was applied to a hightrap heparin prepacked column (GE Healthcare) pre-equilibrated with 25 mM MES buffer containing 1 mM EDTA at pH 6.0 and protein was eluted with 50 mM NaCl in equilibration buffer. The eluted protein was dialyzed against 50 mM Na/Bicine buffer containing 50 mM NaCl at pH 8.3. The sample was further purified by using anion exchanger Mono Q HR 5/50 column (GE Healthcare) pre-equilibrated with 50 mM Na/Bicine at pH 8.3. The protein was eluted using a linear gradient of 0–1.0 M NaCl with 50 mM Na/Bicine buffer. The overall protein purification procedure took approximately 3–4 days.

The purity of the protein was determined by high resolution gel filtration by using Superdex 200 10/30 GL Tricorn column connected to Äkta Explorer purification system (GE Healthcare) as well as SDS-PAGE analysis. The ratio Abs_280_/Abs_450_ was measured to estimate the protein to flavin ratio (PFR) to determine the purity of protein preparation [1], [20]. The concentration of XOR was determined by using an extinction coefficient of 36,000 M^−1^ (subunit) cm^−1^ at 450 nm [1], while total protein was estimated by Lowry's method [34]. The protein was also confirmed by western blotting by using rabbit anti-bovine xanthine oxidase horseradish peroxidase conjugated polyclonal IgG (Pierce product# PA1-46366, Thermo Scientific) and substrate 3-3′-diaminobenzidine tetrahydrochloride (Bangalore Genie, India). The protein bands corresponding to molecular weight of 150 kDa and 123 kDa were further subjected to protein mass fingerprinting by using ESI-LC-MS. XOR fractions were pooled, concentrated and dialyzed against 50 mM Na/Bicine buffer and stored at −20°C in aliquots until further use. The enzyme activity was evaluated to check degradation in the XOR preparation due to storage before performing further analysis. Samples showing decrease in activity were discarded. All experimental errors reported throughout this study were calculated as standard deviation around mean using *n* number of sample size.

### Enzymatic assays and kinetics

The oxidase activity of XOR was determined by spectrophotometric measurement of the rate of oxidation of xanthine to uric acid at 295 nm by using Cary 100 spectrophotometer equipped with the peltier thermostating accessory. For oxidase activity, assays were performed at 25.0±0.2°C in air saturated Na-bicine buffer, pH 8.3 containing 100 µM xanthine, while for total activity (oxidase+dehydrogenase) assays were performed in the presence of 0.5 mM NAD^+^. The dehydrogenase content of the enzyme preparation was determined from the ratio of oxidase to total activity.

Steady state kinetic studies were carried out to determine *K*_m_ and *V*_max_ values. With xanthine as a reducing substrate, uric acid production was monitored as described above by using molar absorption coefficient (*ε*) of 9.6 mM^−1^ cm^−1^ [35] whereas for XDH activity NAD^+^ was used as a reducing substrate and its consumption was measured at 340 nm using molar absorption coefficient (*ε*) of 6.22 mM^−1^ cm^−1^ [36]. 1.0 U activity was defined as 1.0 µmole of uric acid produced or NAD^+^ consumed per min.

### Antibacterial activity of XOR

To study the antibacterial activity of buffalo and cattle XOR, *DH5α* strain of *E. coli* was used as a model microorganism. Cells grown in LB media were washed with PBS buffer. *E. coli* cells were incubated with 10 µM of pterin and 20 mM of inorganic nitrite for 1 hour at 37°C. After one hour incubation, XOR enzyme was added at various concentrations in different tubes containing the cells with substrates and the mixture was immediately subjected to the anaerobic conditions for 10–15 min. After anerobic conditions, cells were spread over LB plates and incubated for overnight at 37°C, and the colonies formed were counted next day. In order to avoid peroxynitrite scavenging activity of uric acid that is produced by action of XOR on xanthine, pterin was used as a reducing substrate [37].

### Determination of FAD content

XOR was dialysed overnight in 50 mM potassium phosphate buffer, pH 7.4. For determination of FAD in various batches of purified XOR, replicates of samples at 552, 312 and 90 nmol subunits of XOR were mixed in 5% (w/v) tricholoroacetic acid (Sigma Chem. Co., USA) and incubated for 30 minutes at 4°C followed by centrifugation at 13,000×g for 10 minutes at 4°C. The fluorescence intensity of the clarified sample was measured at 525 nm with excitation at 450 nm by using the F-6200 spectrofluorimeter (JASCO, Japan). The FAD content in XOR was estimated by using the standard curve of FAD disodium salt (Sigma Chem. Co., USA) as described previously [38].

### Determination of molybdenum content in XOR

Colorimetric assay for determining Mo content in purified XOR was performed after wet-ashing, using a scaled-down method of Hart *et al.* [39] with minor modification and as described by Godber *et al.* [19]. Molybdenum content was determined in different preparation of XOR. To determine content of desulfo fraction of XOR, resulfuration of purified XOR was carried out by incubating the protein preparation with methyl viologen and sodium sulfide as described by Wahl and Rajagopalan [40]. The amount of desulfo form of XOR that got converted in to Mo-sulfo form was calculated from the net increase in the XO specific activity, while the total content of desulfo form of XOR in the initial enzyme preparation was estimated by assuming 50% efficiency of the resulfuration reaction [41].

### Absorbance and circular dichroism (CD) spectroscopy

UV/visible spectra of enzyme samples in 50 mM potassium phosphate buffer (pH 7.4), were recorded on a Cary 100 spectrophotometer. Far UV CD spectrum was measured on Chirascan spectropolarimeter (Applied Photo Physics, UK) by using a quartz cuvet of 1.0 mm path length. Eight CD scans in the 190–260 nm range were accumulated and averaged out followed by conversion to molar ellipticity. Secondary structure composition was evaluated by using the online K2D server [42] by inputting the molar ellipticity values of XOR between 190–260 nm wavelengths. The CD spectrum in the visible region was collected on J-815 CD machine (JASCO, Japan) as well as Chirascan using a quartz cuvet of 10 mm path length using different batches of purified XOR. The content of Fe/S in the XOR was calculated by using the intensity of CD band around 450 nm [19].

### Cloning and sequencing of XOR cDNA

Buffalo mammary gland tissue was obtained from Ghazipur slaughter house, New Delhi, India by written request for use of sample for research purpose. The sample was collected from the slaughtered animal and no animal was slaughtered specifically for this purpose. Total RNA was extracted from tissue of two different animals and cDNA was synthesized by using AccuScript *PfuUltra*II RT-PCR kit (Agilent Technologies, USA). A set of primers (forward: 5′-GCATGAGAGTCCTGTTCCACC-3′ and Reverse: 5′-GGGCAATTCCATCTTCCACG-3′) were constructed from the non-coding region flanking the open reading frame (ORF) of cattle XOR (accession No. NM_173972.2). The amplified PCR product was cloned into pJET 1.2 PCR cloning vector (Fermentas, Lithuania) and transformed in to chemically competent TOP10 *E. coli* strain (Invitrogen Inc., USA). Positive clones were confirmed by PCR amplification of the cloned product. In case of animal 1, only 2.2 kb fragment from 3′end of the ORF could be amplified using a set of internal forward primer and the reverse primer from untranslated region of 3′end. In case of animal 2, we were able to clone complete ORF by using primers constructed from 5′and 3′untranslated regions. Two XOR clones from each of the animal were sequenced and assembled in to a single contiguous consensus sequence. Sequence analysis was carried out by using CLC Main Workbench software (CLC Bio, Denmark).

### Homology modelling

The buffalo XOR model was built using methods and protocols described by Krieger *et al.* [43] by using the Yasara program. The template structures were searched by running six iterations of PSI-BLAST. Five alignment variations per template were allowed for finding the best template and alignment. In some cases only a single model was created for a template if alignment was certain, while in cases where alignment was ambiguous, alternative models were also created. For the top scoring 8 templates, a total of 15 models were created. Based on quality score of 1.0, sequence identity of 97.4% and 100% sequence coverage, the cattle XOR bound with urate (PDB ID: 3AMZ solved at 2.1 Å) with a single unambiguous alignment with the buffalo XOR sequence was selected as a template. The initial model was created as a homodimer of XOR similar to that present in the 3AMZ template. The missing loops were built and the side-chain rotamers were optimized for all the residues by taking in to account electrostatic interactions, knowledge-based packing interactions and the solvation effect [43]. Ligands like molybdopterin, FAD, Fe/S as well as urate present in the template were copied, parameterized and fully included by considering hydrogen-bonding and other interactions with the peptide chain during energy minimization and model building. Distance restraint was used for interactions involving metal ions like Fe/S clusters, while all other ligands were parameterized with AMBER03/GAFF and fully included in the refinement of the model. These methods are implemented in the Yasara-structure software (www.yasara.org) and described elsewhere [43]. The model was refined by using YASARA2 force field that included knowledge-based potentials and parameterized for the refinement of homology models [43]. In the first cycle, energy minimization was carried out with combined steepest descent and simulated annealing by fixing the backbone atoms of the aligned residues to avoid potential damage to the initial model (half energy refined), which was followed by a full unrestrained all-atom simulated annealing minimization. The energy minimization first used implicit solvent during side-chains and loop optimization, while in the simulated annealing minimization explicit solvent shell was used for fine tuning the model. The molecular dynamics (MD) was carried out for refining both the half refined as well as fully refined models in explicit solvent with 0.9% NaCl. The periodic cell contained around 2,22,000 atoms, which included a dimer of XOR with all ligands (one FAD, two 2Fe-2S, one Moco, one calcium ion, one glycerol and one urate per subunit of XOR) similar to that present in the 3AMZ template, water molecules and NaCl. The MD was run for 500 psec with a time step of 2 fsec and saving the trajectory coordinates every 25 psec. Thereafter the root mean square deviation (rmsd) of each of the structure was calculated with respect to the starting model of buffalo XOR. The quality of the model structure was evaluated by considering the overall Z-score which included dihedral angles, planarity and 3D packing terms. The Z-score has been defined as the weighted averages of the individual Z-scores using the formula, overall Z-scores = 0.145×Dihedrals+0.390×Packing1D+0.465×Packing3D that describes how far away is the quality of model in term of standard deviations from the average high-resolution X-ray structure [43]. The negative value suggests that the homology model looks worse than a high-resolution X-ray structure. The quality Z-score was inspected for individual residues as well as for the complete molecule. The detailed stereochemical quality checks of the model were carried out by using Procheck program (version 3.5) prepared by Bernhard Rupp of the Lawrence Livermore National Laboratory for running under Windows NT environment [44].

## Results

### Purification of buffalo milk XOR

**Figure 1:**
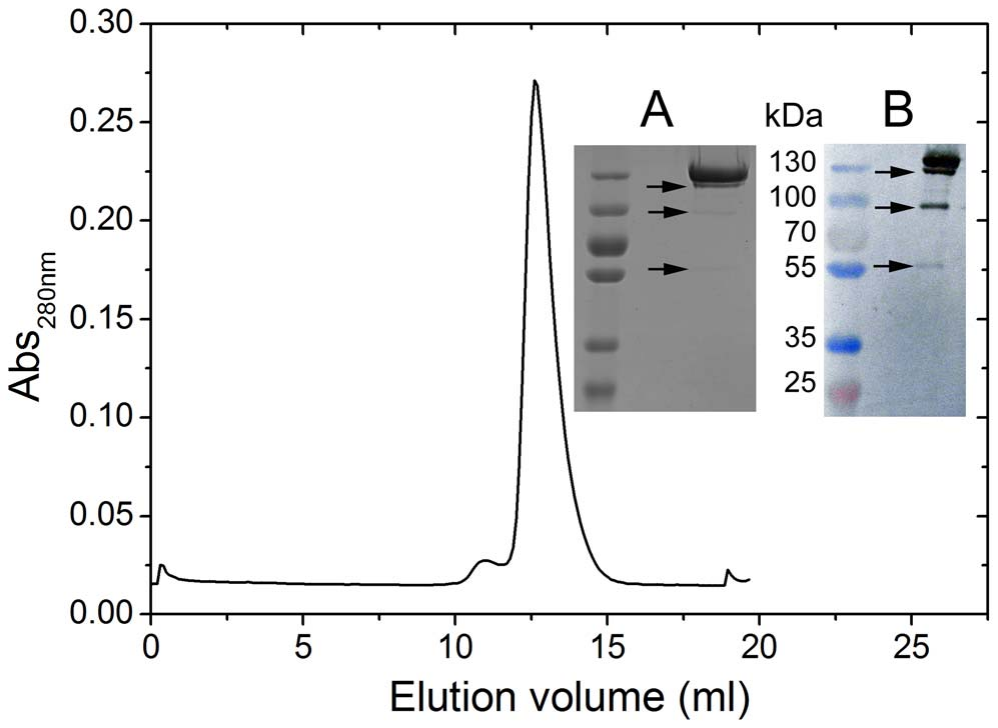
Gel filtration chromatogram of buffalo XOR. 200 µl of MonoQ HR (5/50) purified XOR sample was loaded on high resolution Superdex 200 10/30 GL Tricorn column pre-equalibrated with 50 mM Na/Bicine buffer containing 150 mM NaCl at pH 8.3. The elution was carried out at a flow rate of 0.3 ml/min. The inset Panel A shows the SDS-PAGE (10%) separated proteins obtained from Superdex 200 column, while inset Panel B shows the western blot of same sample. The number in between the panels shows the molecular weight of prestained marker proteins (Thermo Scientific, catalogue No. 26619) electrophoresed in 1^st^ lane on left side of gels. The arrows indicate the position of minor protein bands detected at 123 kDa, 84 kDa and 63 kDa positions on SDS-PAGE and western blots.

The MonoQ anion exchanger purified fraction of buffalo XOR showed a single major peak on a high resolution gel filtration column (Figure 1). The eluted fraction from gel filtration showed a major protein band of molecular weight of 147 kDa and three other minor bands of approximately 120–125 kDa, 80–90 kDa and 60–65 kDa on SDS-PAGE (Figure 1 inset). The two lower molecular weight bands although were very faint. The western blot also confirmed three minor bands (Figure 1 inset) in addition to the major 147 kDa band. It has been known that cattle XOR may undergo proteolytic cleavage at Leu219 and Lys569 during purification [45]. The corresponding positions are Leu220 and Lys569 in buffalo XOR. Complete proteolytic cleavage at these positions in buffalo XOR could result in the conversion of 147 kDa polypeptide chain in to three fragments of molecular masses of 24 kDa, 39 kDa and 84 kDa. However, we could not observe 24 kDa and 39 kDa bands on SDS-PAGE or western blot. The lower two bands observed on SDS-PAGE (Figure 1 inset) were very faint, which suggested that proteolysis should be very mild and only partial. Under the mild proteolytic conditions, partial cleavage at positions Leu220 and Lys569 could result in the generation of 24 kDa/123 kDa fragments, and 63 kDa/84 kDa fragments, respectively. We could detect only 63 kDa, 84 kDa and 123 kDa fragments, while the smaller 24 kDa fragment could not be detected on SDS-PAGE by Coomassie blue staining or by western blotting.

Figure 1 shows gel filtration chromatogram with one major peak centred at 12.5 ml, while smaller fragments (63 kDa, 84 kDa and 123 kDa) revealed on SDS-PAGE were not observed in gel filtration chromatogram. This suggested that under non-denaturing conditions the molecules which underwent proteolysis might remain in a bound state. The homodimers of XOR might be existing apparently as heteromers of cleaved fragments of XOR subunits. The cleavage sites are present in the loop structures connecting Fe/S domain with the FAD domain (Leu220) and FAD domain with the Moco domain (Lys569). Mild proteolysis at these sites should not affect cofactor occupancy or overall composition of the enzyme, although cleavage at Lys569 could result in the conversion of XDH to XO [45]. XO formed by mild proteotytic action should remain in a biologically active dimer of apparent molecular mass almost similar to intact XOR.

The yield of XO activity after butanol treatment of milk fat cream was 85–88%, while around 75–83% with 5–7 fold purification at the end of sequential ammonium sulphate precipitation and dialysis. The yield of XO activity after purification on heparin-agarose column was 32–34% with a fold purification of ∼33–35. After MonoQ step the total activity recovery was 20–25% based on two trials. Purification from three trials from three different animals resulted in an overall yield of 17±0.4 mg of XOR per litre of milk. In case of cattle, using similar protocol the purified protein yield has been reported to be ∼15 mg/L [33]. The peptide mass fingerprinting analysis of 147 kDa and 123 kDa proteins generated significant Mascot score for matching with XOR from other species, which indicated the authenticity of purified buffalo XOR protein. For the sake of comparison of protocols and methods followed in this study, we also purified XOR from Indian cattle milk.

### Activity of buffalo milk XOR

The buffalo XOR showed 0.75±0.04 U/mg of XO activity (*n* = 8) and 0.2±0.03 U/mg of XDH activity (*n* = 6) (Table 1). Since freezing and thawing are known to degrade enzyme preparation, we determined the activity of the XOR preparation before and after thawing the samples. Our results indicated XO activity to be 0.75±0.03 U/mg (*n* = 5) before freezing and 0.73±0.04 U/mg (*n* = 3) after the first thaw. Activity significantly decreased during subsequent freeze and thaw cycles, and therefore the samples were discarded. In case of indigenous cattle, XO and XDH activities were observed to be 1.68 U/mg and 0.25 U/mg. Godber *et al.* [19] reported XO activity of 1.4 U/mg, whereas Benboubetra *et al.* [18] reported a value of 1.8 U/mg for cattle milk XO. Our results on cattle XO are consistent with that reported by other workers, however more closure to that obtained by Benboubetra *et al.* [18]. The XO activity of buffalo XOR was observed almost half that of cattle XOR by using identical purification method. On the other hand, buffalo XDH activity (0.2 U/mg) was similar to that observed for other species [18], [20].

**Table 1:** Comparison of molecular properties of buffalo and cattle milk XORs.

Parameters	Buffalo*	Cattle*
**Molecular weight in kDa**	147	147
**Protein: Flavin ratio (A_280_/A_450_)**	5.1–5.3	5.0–5.2
**XO activity**		
***V*** **_max_** (µmoles/min/mg)	0.75±0.04†	1.68±0.02
***K*** **_m_** (µM)	18.4±1.51	3.6±0.6 [33]
***k*** **_cat_** (1/sec)	2.08±0.04	4.6±0.02
**XDH activity**		
***V*** **_max_** (µmoles/min/mg)	0.2±0.03	0.25±0.05 [18]
***K*** **_m_** (µM)	7.0±1.13	2.75±0.12 [18]
***k*** **_cat_** (1/sec)	0.55±0.03	0.69±0.05 [18]
**Mo (atoms/subunit)**	0.31±0.02	0.58±0.04
**Mo = S (percent)**	52.1±4.0	51.0±2.37
**FAD (atoms/subunit)**	0.99±0.13	1 [19]
**Fe/S I percentage saturation**	77.2	85 [19]

* Data where reference is not cited were obtained in the present study.

† The data have been shown as mean ± standard deviation. The number of experimental replicates have been shown as *n* in the text.

**Figure 2:**
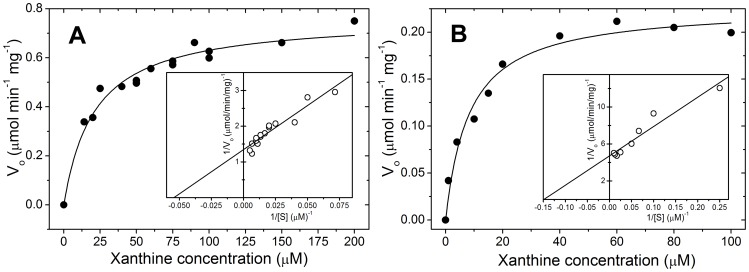
Michaelis-Menten kinetics of buffalo milk XOR for the conversion of xanthine to uric acid. Panel A shows the Michaelis-Menten and Lineweaver–Burk plots (inset) for XO activity in the air saturated reaction buffer and, Panel B shows corresponding plots for XDH activity in the presence of NAD^+^ in the reaction mixture.

The rate constant, *k*_cat_, and Michalis-Menten constant, *K*_m_, values for the conversion of xanthine to urate by buffalo XO were determined to be 2.08±0.04 sec^−1^ (*n* = 3) and 18.4±1.51 µM (*n* = 3) from the MM and LB plots (Figure 2). Previously a *K*_m_ value of 50 µM was reported for buffalo XO [31]. The *K*_m_ value obtained by us was closer to that determined for cattle XO value of 12 µM [46]. Although Godber *et al.* [33] determined a *K*_m_ value of 3.6 µM of xanthine for cattle XO. Other workers have reported *K*_m_ value ranging from 2.15 µM for cattle milk XOR to 6.33–7.74 µM for goat, sheep and human XOR [18]. In our case, the *K*_m_ value obtained for NAD^+^ was 7 µM. For cattle, the *K*_m_ value of XDH has been reported to be ∼2.75 µM, whereas for sheep and goat, the *K*_m_ values lies in the range of 2.1–4.1 µM [18]. The kinetic parameters for XDH activity are shown in Table 1.

### Antibacterial activity of buffalo milk XOR

**Figure 3:**
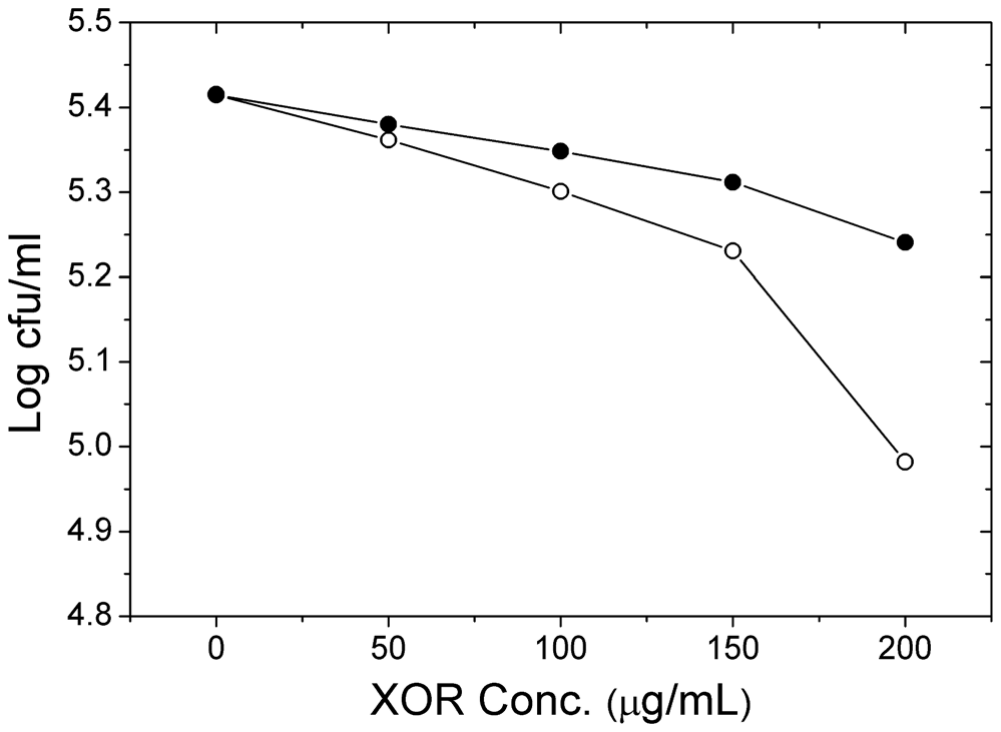
Antimicrobial activity of XOR. The open symbol (○) indicates cattle XOR activity whereas solid symbol (•) indicates buffalo XOR activity.

Figure 3 shows the antibacterial activity of buffalo and cattle XOR. Up to a concentration of 150 µg/mL XOR, the log (CFU/ml) decreased linearly followed by a sharp decrease at 200 µg/mL XOR. At all concentrations, the cattle XOR showed higher bactericidal activity as compared to buffalo XOR. At a concentration of 200 µg/mL, the bactericidal activity of cattle XOR became very significant while buffalo XOR was still following a more or less linear trend. The highest concentration of 200 µg/mL (∼0.67 µM of functional dimer unit) of cattle XOR decreased bacterial count by 0.43 log (CFU/ml), while buffalo XOR could decrease count by only 0.17 log (CFU/ml). The antimicrobial activity of XOR originates from its ability to generate reactive oxygen species (ROS) and reactive nitrogen species (RNS) which could serve as bactericidal or bacteriostatic agents [6]. The results suggest that a threshold level of these radicals might be required to be effective against bacteria. Figure 3 shows that the threshold for antibacterial activity could be 150 µg/mL (0.5 µM) cattle XOR (*n* = 3) and 200 µg/mL (0.67 µM) buffalo XOR (*n* = 3). These results are consistent with the fact that cattle XOR is catalytically more active and hence a stronger producer of free radicals as compared to buffalo XOR.

### Structural analysis of buffalo XOR

#### FAD and Fe/S Centres

**Figure 4:**
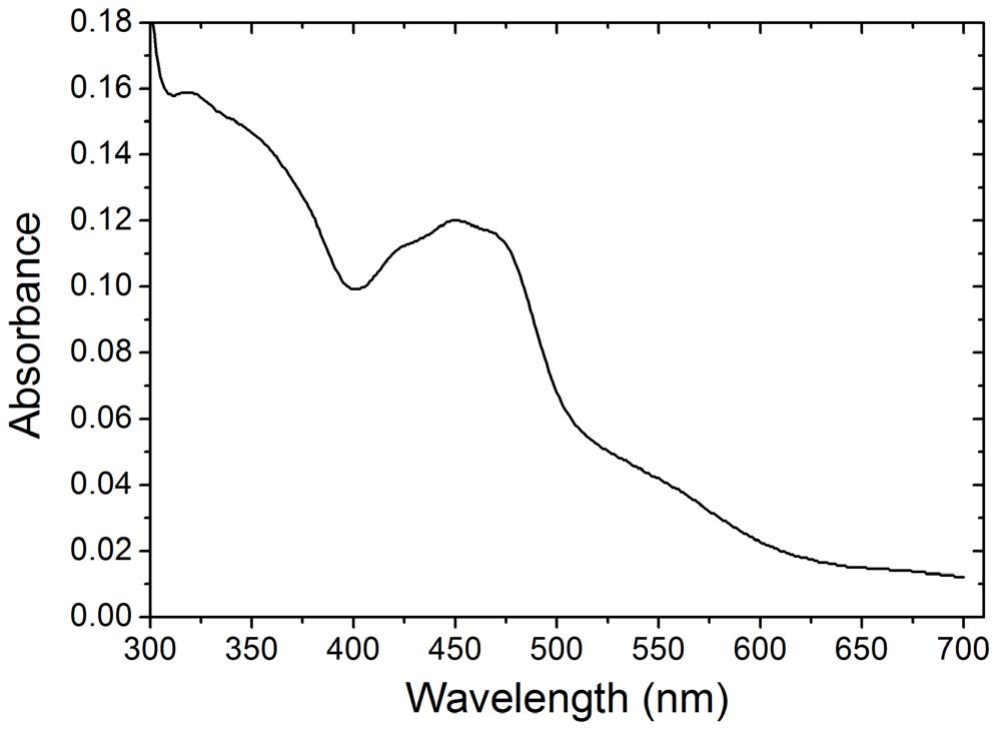
Absorption spectrum of buffalo milk XOR. The peak at 450/S centres, while peak at 550 nm comes from Fe/S centre.

**Figure 5:**
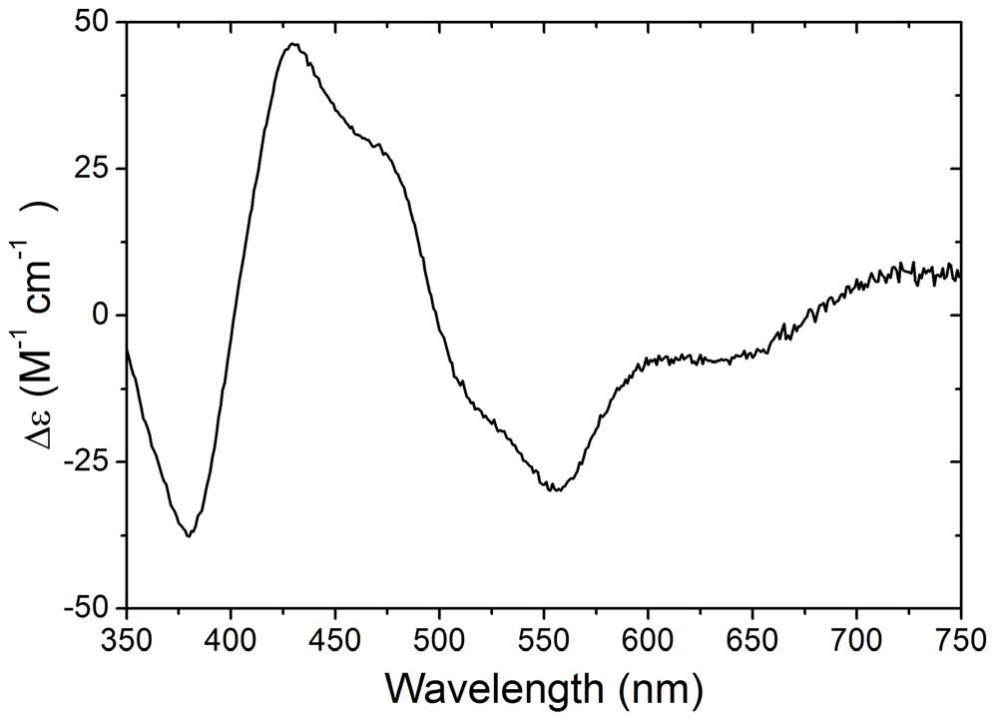
Near UV/visible CD spectrum of buffalo milk XOR. The CD spectrum has been normalized on the basis of FAD content to subunit concentration of 1.0

The content of FAD in XOR was determined to be 0.99±0.13 mol/subunit of XOR (*n* = 6), which suggested one FAD molecule per subunit of XOR. The absorption spectrum of XOR showed two peaks centred at 450 nm and 550 nm (Figure 4). The absorption band at 450 nm has been proposed to arise because of FAD and Fe/S, while weaker absorption band at 550 nm has been solely assigned to Fe/S centres. On the other hand, the positive CD band in the 450 nm region has been ascribed to Fe/S centres [19] and can be used to calculate the occupancy of the Fe/S centres. The CD spectrum of buffalo XOR (Figure 5) closely resembled the CD spectrum of cattle [19] in the 300–660 nm region, which suggested the microenvironment of Fe/S and FAD centres to be similar in XOR from buffalo and cattle. We observed a peak CD value (Δε_430 nm_) of 46,000 M^−1^ cm^−1^ for buffalo milk XOR that translated to an Fe/S occupancy of 88.7% based on Δε_430 nm_ versus Fe/S content in milk XOR from cattle and human [19].

#### Molybdenum cofactor (Moco) centre

The molybdenum content in buffalo XOR was estimated to be 0.31±0.02 (*n* = 9) Mo atom per subunit of XOR (31% saturation). The molybdenum in XOR molecules may exist in the active thio-form [Mo = S] or in the inactive oxy-form [Mo = O] [47]. Therefore, it was crucial to determine the content of catalytically active and inactive forms of Mo in buffalo milk XOR to understand the basis of variation in XOR activity. The theoretical limit of activity-flavin ratio (AFR), which is the maximum XO activity when 100% of Mo is present in the thio-form, to FAD content (Δ*A*_295 nm_ per minute/*A*_450 nm_) has been extrapolated to 210 [1]. For buffalo XOR, we observed an AFR of 105.4, which was equivalent to 50.2% of Mo in the active sulfo form. The resulfuration of buffalo XOR with dithionate over a period of 5 hr incubation resulted in the XO activity increasing to 146±7% of the initial activity. Assuming that sulfuration reaction was only 50% efficient [41], the initial active sulfo form should be 52.1±4% of the total Mo content in buffalo milk XOR. The experimental value was close to the predicted value of 50.2% determined by AFR. Using the experimental value of 52.1% sulfo form of total molybdenum, the net active form of XOR (Mo atom/subunit x sulfo content in %age) should be 16.2% of the total milk XOR. In case of cattle, the XO activity after sulfuration increased to 148.2±4.5%, which translated in to 51±2.4% sulfo form in the native preparation. Other workers have reported 60% [18] and 51.5% sulfo form [19] in cattle milk XOR. Our results provided the net active form of cattle XO to be 29.6% of the total XOR present in milk.

#### Sequence analysis

The 4133 bp cDNA was sequenced and assembled in to a single contiguous sequence and full length ORF (Accession No. JF423940) of 3999 bp was predicted. The alignment of the predicted amino acid sequence of buffalo XOR indicated a similarity of 97.4%, 95.5% and 89% with XOR sequence from cattle, goat and human, respectively (Table 2). There was an insertion at position 189 (Gln189) in buffalo sequence, which was compensated by insertion of Val at position 449 (Val449) in cattle sequence, thereby leaving the total protein length to 1332 residues. This caused a change in the residue numbering for residues within the boundary residues 188–450. XOR is a protein of 1332 residues and divided into three main domains, viz. N-terminal Fe/S domain (residues 3–165) followed by FAD domain (residues 226–531) and C-terminal molybdenum cofactor (Moco) domain (residues 590–1331) [45]. Data shown in Table 2 suggested that pair-wise sequence similarity of Fe/S domain among various species was significantly greater than the overall similarity of full length XOR. The similarity of Moco domain was marginally higher than the overall XOR similarity, whereas similarity of FAD domain was significantly lower (ca 4%) than overall XOR similarity among these species. Variation of one amino acid in Fe/S domain, 14 in FAD domain and 19 in Moco domain of buffalo XOR in comparison to respective domains of cattle XOR were observed.

**Table 2:** Percent sequence identity of various domains of buffalo XOR with goat and human XOR at amino acid level.

Species	Fe/S Domain	Moco Domain	FAD Domain	Overall
**Cattle**	99.4	97.7	95.1	97.4
**Goat**	99.4	95.2	87.6	95.9
**Human**	95.2	91.1	87.6	90.2

Variations between XOR of buffalo and cattle at 14 positions were mapped to helical regions, 10 to *β*-sheets and remaining 10 to random coils (Table 3). Eleven changes took place from a hydrophobic group to another hydrophobic group, e.g. Ile↔Val or Val↔Ala, while 3 changes were polar to another polar groups, e.g. Gln↔His, Ser↔Asn or His↔Tyr, and 7 changes were from charged group to polar group, which involved either isosteric changes like Asp↔Asn, Gln↔Glu or changes among non-isosteric groups like Lys↔His, His↔Asp, Gln↔Arg or Thr↔Lys. Some changes like Glu↔Asp or Lys↔Arg were also observed. The variations among physicochemically unrelated groups like hydrophobic to charged or polar groups or *vice versa* were observed for Ala304↔Ser305, Phe327↔Ser328, Ile407/409↔Lys408/Arg410, Lys472↔Ser472 and Gly1323↔Glu1323. Other two significant changes involved charge inversion, e.g. Lys450/Lys973↔Glu450/Glu973. In the above conversions, the residue shown on left side belongs to cattle, while that shown on right side belongs to buffalo. The change in numbering between cattle and buffalo residues in some cases is because of insertion/deletion events as mentioned above.

**Table 3:** Amino acid variations in various domains of buffalo and cattle XOR.

Sr. No	Residue number & Domain	Buffalo	Cattle
	**Fe/S domain**		
1	141	N	D
	**FAD domain**		
2	305/304†	T	A
3	328/327	S	F
4	339/338	S	A
5	341/340	R	K
6	349/348	I	L
7	406/405	C	L
8	408/407	K	I
9	410/409	R	I
10	424/423	H	Q
11	426/425	N	S
12	450	E	K
13	471	E	Q
14	472	S	K
15	530	E	D
	**Connecting loop**		
16	552	D	H
17	558	R	Q
	**Moco domain**		
18	679	Q	E
19	684	A	V
20	736	V	I
21	752	V	I
22	855	K	T
23	856	V	I
24	944	R	K
25	946	L	M
26	973	E	K
27	1090	I	V
28	1203	M	L
29	1220	Y	H
30	1270	I	V
31	1287	D	N
32	1320	C	G
33	1321	V	A
34	1323	E	G

† Different residue numbering has been shown where buffalo and cattle XOR residues differed because of insertion/deletion events. The first number belongs to buffalo XOR residue while second number belongs to cattle XOR residue.

#### Secondary structure analysis

Deconvolution of circular dichroism (CD) spectrum of buffalo milk XOR in the far-UV region (190–260 nm) provided 36% *α*-helix, 21% *β*-pleated sheets and 43% random coil content, which was consistent with the secondary structural composition of cattle XOR determined by X-ray crystal analysis [45].

#### Structural modelling

Buffalo XOR structure has not been solved. Using the cattle xanthine oxidase structure as a template, the buffalo homology model was built to observe the impact of sequence variation on the structure and function of XOR. Three loop structures (residues 166–192; 529–538; 1320–1326) missing in the 3AMZ cattle template were built in buffalo XOR model. The half energy refined (with fixed backbone atoms) model (Z-score = −0.656) was better than fully refined model (Z-score = 0.680). The obtained Z-score values of the models suggested that the quality of the models is close to that of experimental crystal structure. The quality Z-score value below −2.0 is considered bad. The analysis of 20 snapshots saved during 500 psec molecular dynamics trajectory of both the structures, i.e. initial half refined and fully refined models, suggested that the structures deteriorated in comparison to the respective initial models as indicated by an increase in their rmsd and a decrease in the quality Z-score values (data not shown). The stereochemical quality check of the half refined buffalo XOR model (1332 total residues) showed 90.6% residues falling in the most favoured regions, 8.8% in the additional regions and 0.3% in the generously additional regions, while the corresponding values for the experimentally determined template cattle XOR structure were 90.2, 9.1 and 0.2, respectively, for 1292 residues, since several loop regions were missing in the cattle XOR template structure. The overall average G-factor for buffalo XOR calculated by Procheck program was better at 0.16 as compared to −0.09 for cattle XOR. Buffalo XOR model did not show any clash or bad contact. These analyses suggested good overall stereochemical quality and quality Z-score of the half-refined buffalo XOR model. Therefore, we used the half energy refined structure for further analysis.

**Figure 6:**
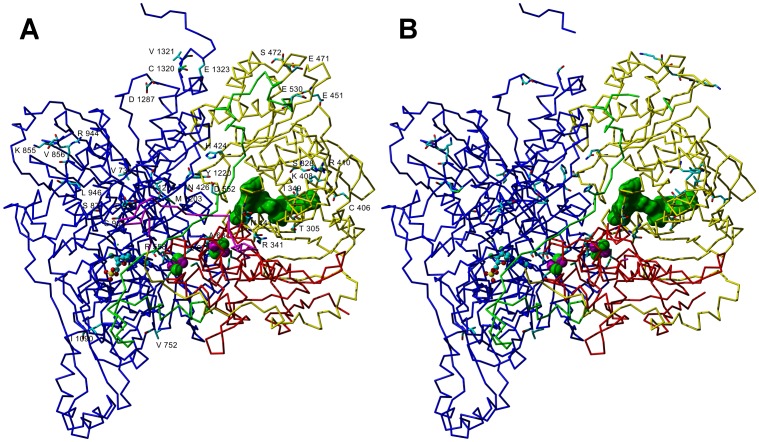
The α-carbon trace models of monomeric XOR. Panel A shows buffalo XOR while Panel B shows cattle XOR. The solid green color surface indicates the FAD molecule, the two 2Fe-2S (Fe/S) cofactor have been shown in space filling atomic representation in green (sulfur) and magenta (iron) color, while the Molybdenum cofactor (Moco) has been shown in ball and stick representation. The magenta color loop in buffalo XOR model, which is absent in electron density map of template cattle model (PDB ID: 3AMZ), connects the Fe/S domain (red color) with FAD domain (yellow color). The extended loop (residues 528–589) shown in green color connects the FAD domain with Moco domain (blue color). The residues shown with labels in buffalo XOR (Panel A) are only those which differed from corresponding residues in cattle XOR (Panel B) and also shown in Table 3. In case of template cattle XOR model, several loop structures were missing, which were built for buffalo XOR as described in the text.

Superimposition of the protein part of the model structure of buffalo XOR subunit over the corresponding template subunit of cattle provided a rmsd of 1.227 Å over a total of 1287 aligned residues found matching in buffalo and cattle XOR structures since three loop structures were missing in the template (Figure 6).

In buffalo XOR the Gln189 was inserted in the extended loop connecting Fe/S domain with FAD domain and shown in magenta color in Figure 6, while Val449 was inserted in cattle XOR in the loop (residue 445–449) connecting two *β*-strands. Residues Phe327, Leu348, Ile407 and Ile409 constituting a highly hydrophobic pocket near FAD binding site in cattle XOR were converted to Ser328, Ile349, Lys408 and Arg410 in buffalo (Figure 6), respectively. Several residues of this pocket directly make contacts with FAD molecule. In this pocket, mutually interacting hydrophobic residues like Phe327 – Ile409 and Leu348 – Ile407 in cattle were replaced by Ser328 – Arg410 and Ile349 – Lys408, respectively, however, in the later case Lys408 changed its orientation to avoid unfavourable interaction with Ile349. It seems that a change at a given position might have induced other changes to accommodate the variant residue to maximize the overall interactions for preserving the stability and folding of the molecule.

**Figure 7:**
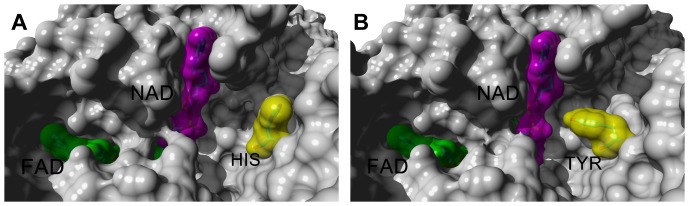
Molecular surface of XOR around FAD reaction centre showing His1220 in cattle XOR and Tyr1220 in buffalo XOR. Panel A shows cattle XOR (PDB ID: 3AMZ) subunit, while Panel B shows model of buffalo XOR subunit. The grey color surface represents the protein molecular surface, the green color surface indicates FAD molecule, while magenta color surface indicates NAD molecule. The yellow color surface indicates the His1220 in cattle XOR (Panel A) and Tyr1220 in buffalo XOR (Panel B). The analytical molecular surfaces were created by using the fine grid resolution and the water probe radius of 1.4 Å in Yasara program [43].

Variations at two positions (Gln423↔His424 and Ser425↔Asn426) in the loop spanning residues 423–433, which restrict the entry of NAD^+^ after conversion of XDH to XO [45], were also observed between cattle and buffalo. An important change was observed at the substrate entry site, wherein His1220 as a part of flexible random coil in cattle XOR was replaced by a bulkier tyrosine residue in buffalo. The His1220 in cattle was flipped away from NAD^+^ binding pocket, while in case of buffalo Tyr1220 projected towards NAD^+^ and occupied the pocket (Figure 7). In cases where xanthine oxidase template was used for structural modelling of buffalo XOR, the Tyr1220 was shifted to a similar position as occupied by His1220 in cattle XO due to the large scale movement of the loop (residues 423–433) in the active site after conversion of XDH to XO form (data not shown). It is quite possible that Tyr1220 may sample both the positions in buffalo XDH form.

Another important change was the presence of Asn1287 in cattle and Asp1287 in buffalo XOR. Asp1287 in buffalo XOR can form extensive network of interactions with nearby Arg1279, Arg1282 and Glu1292, whereas Asn1287 in cattle XOR can form network of interaction with only Arg1282 and Glu1292.

## Discussion

The XOR enzyme was purified from milk using well established method [19], [33]. The most crucial step involved use of butanol to release XOR from MFGM. Because of hydrophobic nature butanol could denature proteins if proper temperature is not maintained, therefore pre-chilled butanol at −20°C was essentially added at slow rate resulting in the recovery of XO activity as high as 85–88% at the end of this step. This suggested that butanol treatment was very mild and had no significant effect on protein structure and activity. It was necessary to ensure the consistency of the methods as well as that XOR was not denatured that could have affected the analysis of biochemical properties and cofactor content. Analysis of secondary structure of buffalo XOR by using CD spectrum in the far UV region showed almost similar content of secondary structural elements as has been known of cattle XOR from x-ray structure [45]. The CD spectrum in the visible region (Figure 5) also clearly suggested the environment of Fe/S and FAD intact and very similar to that obtained for cattle XOR [19]. The protein to flavin ratio (PFR) of 5.1–5.3 (Table 1) was consistent with earlier studies on XOR from other species. The content of FAD was 1 FAD/subunit of XOR, had there been any denaturation of XOR, the content of FAD per subunit would have accordingly changed. These facts clearly suggested that protein preparation was of good quality and did not suffer with any experimental artifact like protein denaturation that could have altered cofactor composition. Moreover, we also purified cattle milk XOR and determined PFR, molybdenum and sulfurated molybdo contents, which were similar to that reported in other studies. These results clearly suggested that the employed purification and other methods were consistent and did not introduce any artifact that could affect our analysis of cofactors or biochemical properties presented in this study.

Wide variation in the XO activity has been observed among various species despite high sequence similarity. Cattle XO activity was observed 2.25 times higher than that of buffalo XO activity, which in turn was 2.7 times greater than goat XO activity. These ruminant species showed XOR sequence identity of >95% at amino acid level. The shape of the absorption and CD spectra of buffalo milk XOR were similar to that of cattle XOR; however minor changes in CD peak height in the 350–650 nm region suggested altered composition of cofactors. CD spectrum in the far UV region suggested similar content of secondary structural elements, which was also supported by the homology modelling of buffalo XOR. Many of the variations seem neutral as they involve either isosteric changes or conversion among physicochemically similar residues. These changes are known to preserve the structure, however they might cause change in stability of proteins [48]. Active site residues like Gln767, Glu802, Arg880, Phe914, Glu1261 were conserved between cattle and buffalo suggesting that these residues may not be involved in activity variation between cattle and buffalo XORs. On the other hand, the buffalo XOR model showed some crucial differences, especially at the substrate entry site of the FAD domain, where His1220 in cattle XOR was replaced by Tyr1220 in buffalo XOR (Figure 7). Buffalo XOR also differed from cattle XOR at several positions in close proximity (6–10 Å) of FAD molecule (Figure 6). In comparison to cattle, two variations at positions 424 (Gln→His) and 426 (Ser→Asn) were also observed in the loop (residue 423–443) that has been reported to cause steric hindrance for the entry of substrate NAD^+^ in the FAD reaction centre [45]. The FAD domain was more resilient to sequence variation as compared to Fe/S and Moco domains and these variations did not lead to any change in the occupancy of FAD site. Even though such variations cumulatively might affect the functionality of the enzyme, but no direct correlation could be derived between alteration of XOR structures and more than two fold variation in the XO activity. These results suggested that the variation in xanthine oxidase activity could be originating from other than protein structural factors to a large extent. Therefore, we analysed the occupancy of molybdenum, Fe/S and FAD centres which are crucial for the intramolecular electron transport (IET) and hence biochemical properties of XOR.

### Iron-sulphur and FAD Centres

Iron-sulphur (Fe/S) centres play an important role in the electron transfer chain and are structurally localized at an intermediate position to receive electron from Moco reaction site for transfer to terminal FAD centre for the reduction of NAD^+^ or O_2_. The deficient Fe/S centre could seriously hamper the electron transfer to FAD centre. Electron paramagnetic resonance studies have shown that Fe/S II centre in cattle XOR has occupancy of 1.0, while Fe/S I centre showed occupancy of 0.85, i.e. 15% Fe/S deficiency [19]. The CD (Δε_430 nm_) value of buffalo XOR showed an overall Fe/S deficiency of 11.3% in buffalo XOR. Assuming the Fe/S II centre to be fully occupied similar to that in cattle, the Fe/S I centre in buffalo XOR should then be deficient by 22.6% in comparison to 15% in case of cattle XOR. On the other hand, human XOR showed Fe/S I centre deficient by 31.3% [19]. Significant difference in the structure around Fe/S centres is not expected given the difference of only one amino acid between the buffalo and cattle Fe/S domains of XOR (Table 3). The Fe/S domain buried in between the two other domains holding Moco and FAD cofactors help in maintaining the scaffold for faithful transfer of the electrons. Given the important structural role played by Fe/S domain in electron transfer, the large scale structural differences might not be tolerable.

FAD is responsible for the reduction of molecular oxygen or NAD^+^ at the terminal step of IET chain. Fully saturated FAD site in XOR from various species indicated that FAD *per se* might not be responsible for the varying XO activity. Interestingly, largest number of variations among the XOR from various species occurred in the FAD domain suggesting that FAD domain is more resilient to sequence variations. Several variations in close proximity of FAD although could impact it's interaction with protein.

### Moco domain and activity variation

The sequence data indicated that Moco domain was conserved to a greater extent than the average overall similarity of XOR among various species. The higher than average conservation of this domain could be because Moco centre constitutes the first step in the IET and it is also involved in the dimerization of the XOR subunits. In spite of high sequence conservation of Moco domain, large scale variations in the occupancy of molybdenum centre were observed among the species. It has been reported that catalytic activity of XOR depends upon the presence of sulfo form of Mo [49] as opposed to oxy form. A major fraction of buffalo milk XOR was observed in demolybdo or desulfo form which rendered almost 84% milk XOR to be catalytically inactive as compared to 70% observed in cattle milk XOR. We observed a *k*_cat_ value of cattle XO to be 2.25 times higher than that of buffalo XO, whereas net active form, i.e. sulfurated molybdo form of cattle XO was 1.83 times higher than that of buffalo. These results suggested that the variation in the content of molybdenum and its sulfo form is the major deciding factor but not the only factor responsible for the variation in the XO activity. Apart from the molybdenum associated inactivity, the remaining difference in the activities between the two species could be originating from factors like deficiency at Fe/S centres. Minor structural variation around the cofactor binding site could also affect cofactor occupancy as well as catalysis event.

### Enzymatic efficiency of buffalo XOR

The XO enzymatic efficiency (*k*_cat_/*K*_m_) of 0.11 sec^−1^ µM^−1^ of buffalo XOR was much smaller than that of cattle's 1.27 sec^−1^ µM^−1^ by a factor of ∼10, which is much larger than the difference in their XO specific activities. This suggested that buffalo milk XO cannot utilize xanthine and molecular O_2_ as efficiently as cattle XO due to higher *K*_m_ value for xanthine. Similarly, the XDH enzymatic efficiency of buffalo XOR was lower by a factor of ∼3 in comparison to that of cattle XDH whereas their specific activities did not significantly differ. This means that buffalo XDH cannot also utilize NAD^+^ as efficiently as the cattle XDH. The comparison of structures of cattle and buffalo XOR suggested that substitution of His1220 in cattle XOR with Tyr1220 in buffalo XOR could be playing a role in ease of accessing FAD centre by NAD^+^, since tyrosine can partially occupy the active site pocket and restrict the entry of NAD^+^ (Figure 7). The proposition, however, needs to be proved by site directed mutational analysis.

It is also possible that electron transport from the Moco reaction centre might be limited for the reduction of NAD^+^ at FAD centre and consequently could affect the rate of reaction when utilizing NAD^+^ as the electron acceptor. This is supported by the fact that similar rates of reactions were obtained when reaction was monitored either by measuring the production of uric acid at Moco reaction centre or the reduction of NAD^+^ at FAD reaction centre. XOR has been shown to possess NAD^+^ reducing activity independent of xanthine oxidation with an intrinsic rate of reaction much higher than the rate of xanthine oxidation [50]. However, in a coupled reaction, the rate of NAD^+^ reduction should be limited by the availability of electrons in the IET chain. It means that the intrinsic rate of NAD^+^ reduction is not a rate limiting step and the lower efficiency of reaction at FAD reaction centre could be due to limited supply of electrons or the restricted access of FAD reaction centre by NAD^+^ in case of buffalo. The later proposition is supported because *k*_cat_ is similar for XDH activity among various species whereas *K*_m_ value for NAD^+^ as a substrate significantly differed between buffalo and cattle XDH (Table 1).

### Physiological significance of high content of inactive XOR in milk

XOR, a major protein component of MFGM, has been implicated in the process of milk lipid secretion [51], [17] and plays an important role in enveloping lipid droplets by the cell membrane by virtue of its structure rather than its enzymatic activity. This could possibly explain the predominance of inactive XOR in milk. On the other hand, fully active XO can create serious problem by producing free radicals in excess leading to tissue injury [47]. Moreover, synthesis of fully active sulfurated molybdo-form of XOR is an expensive process involving conversion of GTP to pterin *via* a complex Moco biosynthesis pathway followed by sulfuration by Mocosulfurase. Limited production of Moco could result in a major fraction of XOR in demolydo and or desulfo-forms. What could then be the role of mixed population of catalytically active and inactive forms of XOR in milk? It has been suggested that microbicidal activity of milk XOR in the neonatal gut could be providing protection against infection during the early phase [5], [20], [37], [47]. Our data show that at higher concentration XOR works as a bactericidal agent. In new-born ruminants, milk directly goes to abomasum where XOR acts as a bactericidal agent and may prevent gastrointestinal tract infection. It is noteworthy that in the first few weeks after parturition both XO [52] and nitrite reductase [4] activities in human milk are much higher than in subsequent fractions. Our preliminary results also suggested higher XO activity in milk and upregulation of mRNA transcripts of some key Moco biosynthesis pathway enzymes like MOCS1 and Mocosulfurase genes in buffalo mammary epithelial cells after two weeks of parturition (unpublished results). This might result in higher proportion of XOR partitioning to active form due to enhanced expression of Moco synthesizing enzymes under the influence of heightened level of lactogenic hormones after parturition. It has been reported that XOR expression remained invariable, while the specific activity increased in the initial phase of lactation [17], [52]. The cofactor-lacking inactive form of XOR on the other hand might facilitate the milk fat globule formation and milk synthesis [14]–[17].

In conclusion, the buffalo milk XOR was observed less efficient than cattle XOR with respect to xanthine oxidation as well as NAD^+^ reduction in spite of very high sequence similarity. Buffalo XOR was observed to possess lower content of sulfurated molybdenum that directly affected catalytic activity in a major way. The weaker XOR activity also resulted in the production of lower free radical activity and hence a lower antibacterial activity as compared to cattle XOR.
